# Nutritional status, weight perception and weight control practices among office employees in Sokoto, Nigeria

**DOI:** 10.11604/pamj.2017.27.279.12222

**Published:** 2017-08-15

**Authors:** Kehinde Joseph Awosan, Semiyu Adetunji Adeniyi, Hamza Bello, Zarau Bello-Ibrahim

**Affiliations:** 1 Department of Community Health, Usmanu Danfodiyo University, Sokoto, Nigeria; 2 Noma Children’s Hospital, Sokoto, Nigeria; 3 1^st^ Brigade Medical Centre, Army Barracks, Sokoto, Nigeria

**Keywords:** Nutritional status, weight perception, weight control practices, office employees

## Abstract

**Introduction:**

Overweight and obesity have become the fifth leading risk for global deaths. Office employees have been identified as a high risk group due to the sedentary nature of their work, and accurate weight perception is believed to be critical to acceptance of weight control interventions. This study was conducted to assess the nutritional status, weight perception and weight control practices of office employees in Sokoto, Nigeria.

**Methods:**

A cross sectional study was conducted among 285 randomly selected office employees in private establishments in Sokoto, Nigeria, in February and March 2013. Anthropometry was done for the participants in addition to questionnaire administration.

**Results:**

The mean age of the participants was 33.08 ± 7.23 years, they were predominantly males (56.5%) and married (57.5%). None was underweight, 111 (38.9%) had normal weight, 105 (36.8%) were overweight and 69 (24.2%) were obese. Among the participants with normal weight, overweight and obesity, 71.2%, 35.2% and 58.0% respectively accurately perceived their weight; while 28.8%, 50.5% and 30.4% respectively underestimated their weight. There was poor agreement between actual and perceived weight (k statistics = 0.341, p < 0.001). Only 67 (23.5%) of the 285 participants were engaged in weight control practices.

**Conclusion:**

This study showed high prevalence of overweight and obesity, weight misperceptions, and poor uptake of weight control practices among office employees in Sokoto, Nigeria. These findings underscore the need for a holistic approach to obesity control interventions that encompasses both body image perception and nutritional assessment.

## Introduction

Once considered a high-income country problem, overweight and obesity are now on the rise in low- and middle-income countries, particularly in urban settings. In 2014, more than 1.9 billion adults, 18 years and older, were overweight. Of these, over 600 million were obese. Overall, in 2014, about 13% of the world’s adult population (11% of men and 15% of women) were obese, while 39% of adults aged 18 years and over (38% of men and 40% of women) were overweight. In Africa, the number of children who are overweight or obese has nearly doubled from 5.4 million in 1990 to 10.6 million in 2014 [1]. The global epidemic of overweight and obesity is believed to be driven by the increased intake of energy dense foods that are high in fat and an increase in physical inactivity due to the increasingly sedentary nature of many forms of work, changing modes of transportation and increasing urbanization across the globe [1].

The Centers for Disease Control and Prevention (CDC) estimates show that more than one third (36.5%) of United States adults have obesity, with the highest age adjusted prevalence in Non-Hispanic blacks (48.1%) [2]. In the United Kingdom and Ireland, about 62.9% of adults were estimated to have either overweight or obesity in 2015 with almost equal prevalence among men and women (67.8% of men and 58.1% of women) [3]. In a systematic review and meta-analysis by Ofori-Asenso et al, it was reported that 43% of Ghanian adults are either overweight or obese, and the national prevalence of overweight and obesity were estimated at 25.4% and 17.1% respectively [4]. Another study in Dar es Salaam, Tanzania, reported 13% and 36% prevalence of obesity in men and women respectively [5]. Reports from studies in Nigeria essentially mirror the situation across the globe. A study by Adedoyin et al, in Ile-Ife, south western Nigeria, reported 20.3% and 35.1% prevalence of overweight and obesity respectively, while another study by Wahab et al, in Katsina, northern Nigeria, reported 53.3% and 21% prevalence of overweight and obesity respectively [6–7].

Overweight and obesity have become the fifth leading risk for global deaths. Most of the world’s population lives in countries where overweight and obesity kills more people than underweight. At least 2.8 million adults die each year as a result of being overweight or obese. Also, 44% of the diabetes burden, 23% of the ischemic heart disease burden and between 7% and 41% of certain cancer burdens are attributable to overweight and obesity [8]. In addition to the high morbidity and mortality from overweight and obesity, individuals with these conditions suffer from adverse psychosocial outcomes including, stigmatization and discrimination, depression, dissatisfaction with body image, poor quality of life, and suicidal ideation or attempts, particularly among women and adolescents [9–13]. Importantly, occurrence of these adverse psychosocial outcomes, and adoption or otherwise of healthy weight control behaviors are largely determined by how individuals perceive their weight, and not their actual weight status. Weight perception (or perceptual body image) constitutes one of the four components of body image; the other components include affectual body image, cognitive body image and behavioral body image [14]. The perceptual body image refers to how one sees his or her body. The perceived body weight status is not always a true representation of the actual body weight status, and so weight misperception is said to occur when there is discordance between an individual’s actual weight and the perception of his or her weight. In the Youth Smoking Survey 2003-2004 among 20,677 normal weight students aged 11-18 years from 85 randomly selected schools throughout Hong Kong, misperceived fatness or thinness was found to be associated with adverse psychosocial outcomes including headache, feeling stressful, depression, poor appetite, insomnia, nightmares, and less confidence in getting along with friends [15].

However, perception of body image is believed to be influenced by social norms and cultures. While a thin body is preferred as ideal body image for females in developed countries, heavier body is preferred in developing countries; and in some cultures particularly in sub Saharan Africa and Asia, moderately fat women are considered to be more attractive, and a sign of affluence [16–19]. The perceived body image invariably determines how individuals feel about their body (this is the affectual body image). It relates to the amount of satisfaction or dissatisfaction one feels about his or her shape, weight and individuals body parts [14]. Cognitive body image refers to the way one thinks about his or her body, it can lead to preoccupation with body shape and weight. For example, some people believe they will feel better about themselves if they are thinner or more muscular; and this eventually determines the weight control behaviors (behavioral body image) they engage in [14]. A positive body image is said to occur when a person is able to accept, appreciate and respect his or her body, and it is of immense benefit in terms of improved self-esteem, self-acceptance and healthy outlook and behavior [14]. Unfortunately, the reverse is true in most cases, due to the prevalent weight misperceptions in different populations across the globe, thus culminating in various adverse psychosocial outcomes and unhealthy body weight control practices [20–24].

Office employees have been identified as a high risk group for overweight, obesity and risk factors of cardiovascular diseases due to the sedentary nature of their work and unhealthy dietary practices [25–28]. On the other hand obesity has been associated with adverse workplace effects including increased rate of absenteeism (i.e., more days out of work) and presenteeism (i.e., reduced productivity while at work). In addition, obese workers are known to take more sick days, have longer sick leaves, and incur greater productivity losses than do non-obese workers [29]. Research exploring the relationships between nutritional status, weight perception and weight control practices among high risk groups particularly office workers is limited in Nigeria. Most of the studies conducted across the country had reported high prevalence of misperception of body weight status and unhealthy weight control practices [30–32]. Accurate perception of body weight status is believed to be crucial to uptake of body weight control interventions [11–20]; this study was therefore conducted to assess the nutritional status, weight perception and weight control practices of office employees in Sokoto, Nigeria. The findings from the study would be invaluable in designing appropriate strategies and interventions for the prevention and control of obesity and unhealthy weight control practices, particularly in populations with similar characteristics worldwide.

## Methods

### Study design and population

This was a cross sectional study conducted among office employees in private establishments in Sokoto metropolis, North Western Nigeria, in February and March 2013. Sokoto metropolis is both the capital and commercial centre of Sokoto state. It contains most of the industries in the state in addition to the Sokoto central market which is one of the largest markets in Nigeria. People from all over the state, the neighboring states of Kebbi, Zamfara and Katsina, and the neighboring country (Niger Republic) come to the city to transact business. Sokoto state has an estimated population of 4,802,298 projected for 2015 based on the 2006 census [33]. The Hausas and Fulanis constitute the predominant ethnic groups, they are mainly farmers but they are also involved in other occupations such as tanning and dyeing. The other ethnic groups in the state include Igbo, Yoruba and Igala among others. Most of them are traders, artisans and civil servants. Employees in the selected private establishments that have worked for one year and above and gave their consent to participate were considered eligible for enrollment into the study. The sample size was estimated at 285 using the formula for proportion [34] 16.3% prevalence of obesity among traders in a previous study [35], precision of 0.05 and response rate of 80%. The eligible participants were selected by multistage sampling technique. At the first stage, Sokoto metropolis was divided into 12 business Areas and 6 were selected by simple random sampling using the ballot option. At the second stage, selection of private establishments in each of the selected districts was done by systematic sampling technique using the list of private establishments in each business area to constitute the sampling frame. Proportionate allocation (based on number of private establishments) was applied in the selection of private establishment in the selected business areas. At the third stage, selection of participants in the selected private establishments was done by systematic sampling technique using the staff list in the respective private establishments to constitute the sampling frame. Proportionate allocation (based on the staff strength) was also applied in the selection of participants in the selected private establishments.

### Data collection

The methods of data collection comprised of questionnaire administration and physical assessment (anthropometry). A standardized semi-structured, interviewer-administered questionnaire was used to obtain information on the socio-demographic characteristics of the study participants, weight (body image) perception and weight control practices. Participants were asked to select the option that best describes how they see themselves out of “too thin”, “normal”, “a bit fat” and “too fat” which represent underweight, normal weight, overweight and obese respectively. The instrument was pre-tested among 20 office workers in one of the wards not selected for the study; the necessary adjustment was effected based on the observations made during the pre-test. Anthropometry (weight and height measurement) was done for assessment of nutritional status of the participants. Weight was measured with shoes off to the nearest 0.5kg using a Seca Optimal scale; it was validated with a standard weight and corrected for zero error. Height was measured without shoes to the nearest 0.5cm using a stadiometer. Five medical officers assisted in data collection after pre-training on the objectives of the study, selection of study participants and use of survey instruments. Ethical clearance was obtained from the Ethical committee of Sokoto state Ministry of Health; permission to conduct the study was obtained from the Management of the respective establishments selected for the study, and informed written consent was also obtained from the participants before data collection.

### Operational definition of terms

Body mass index (BMI) was calculated as weight (kg) divided by height^2^ (m^2^) and used as marker for nutritional status [**36**]. Underweight was defined as BMI less than 18.5kg/m^2^, normal weight was defined as BMI of 18.5 to 24.9kg/m^2^, overweight was defined as BMI of 25.0 to 29.9kg/m^2^, while obesity was defined as BMI of 30.0kg/m^2^ and above.

### Data analysis

Data were analyzed using IBM SPSS version 20 computer statistical software package. Frequency distribution tables were constructed; cross tabulations were done to examine relationship between categorical variables. The Chi-square test was used to compare differences between proportions. Kappa statistic was used to measure the agreement between nutritional status and perceived weight. Logistic regression analysis was used to determine the predictor of accurate weight perception. All statistical analysis was set at 5% level of significance (i.e. p < 0.05).

## Results

The mean age of the participants was 33.08 + 7.23 years, with the largest proportion (41.1%) in the 30 – 39 years age group. Majority of the 285 participants were males (56.5%) and married (57.5%). A larger proportion of the participants had tertiary education (40.7%), followed by those with secondary education (38.6%), while about a fifth of participants (20.7%) had primary education and below (Table 1).

**Table 1:** Socio-demographic profile of participants

Variables	Frequency (%) n = 285
**Age group (years)**	
20-29	100 (35.1)
30-39	117 (41.1)
40-49	53 (18.6)
50 and above	15 (5.3)
**Sex**	
Male	161 (56.5)
Female	124 (43.5)
**Marital status**	
Single	114 (40.0)
Married	164 (57.5)
Separated	5 (1.8)
Widowed	2 (0.7)
**Education level**	
Primary and below	59 (20.7)
Secondary	110 (38.6)
Tertiary	116 (40.7)

### Nutritional status of participants

**Figure 1:**
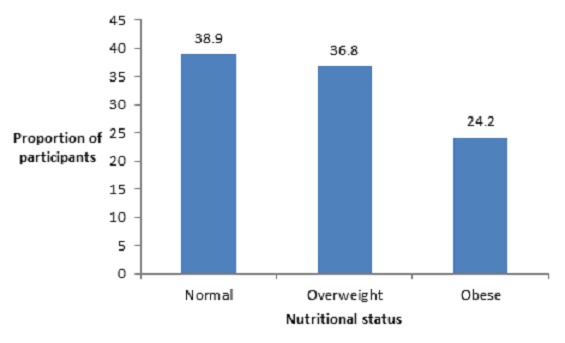
Nutritional status of participants

None of the 285 participants was underweight, 111 (38.9%) had normal weight, 105 (36.8%) were overweight and 69 (24.2%) were obese (Figure 1). While majority of participants in the 20 – 29 years age group had normal weight, overweight was more prevalent among participants aged 30 – 49 years, and obesity was more prevalent among participants aged 50 and above; the differences in the distribution of nutritional status by age group was found to be statistically significant (χ^2^ = 16.996, p = 0.009). Similarly, while overweight was significantly more prevalent among males, obesity was more prevalent among females (χ^2^ = 7.801, p = 0.020). Although there were differences in the distribution of nutritional status by marital status, they were not statistically significant (χ^2^ = 10.077, p = 0.121) as shown in Table 2.

**Table 2:** Distribution of nutritional status by socio-demographic profile of participants

Variables	Nutritional status		
	Normal Frequency (%)	Overweight Frequency (%)	Obese Frequency (%)
**Age group (years)**			
20-29	52 (52.0)	30 (30.0)	18 (18.0)
30-39	45 (38.5)	46 (39.3)	26 (22.2)
40-49	11 (20.8)	25 (47.2)	17 (32.1)
50 and above	5 933.3)	4 (26.7)	6 (40.0)
**Sex**			
Male	60 (37.3)	70 (43.5)	31 (19.3)
Female	53 (42.7)	35 (28.2)	36 (29.0)
**Marital status**			
Single	56 (49.1)	33 (28.9)	25 (21.9)
Married	55 (33.5)	69 (42.1)	40 (24.4)
Separated	1 (20.0)	3 (60.0)	1 (20.0)
Widowed	1 (50.0)	0 (0)	1 (50.0)

### Body image (weight) perception by participants

Table 3 shows the distribution of body image (weight) perception by nutritional status (BMI category) of participants. Among the participants with normal weight, overweight and obesity, 71.2%, 35.2% and 58.0% respectively accurately perceived their weight; while 28.8%, 50.5% and 30.4% respectively underestimated their weight. There was poor agreement between actual and perceived weight (k statistics = 0.341, p < 0.001). However, a few participants with overweight (14.3%) perceived themselves to be obese. There was significant association between normal weight and accurate weight perception (χ^2^ = 45.03, p < 0.001). In logistic regression model, participants with normal weight were thrice more likely to perceive their weight accurately compared with those with overweight and obesity (Odds Ratio = 3.110, 95% CI = 1.871 – 5.169, p < 0.001).

**Table 3:** Distribution of body image (weight) perception by nutritional status (BMI category) of participants

Nutritional status (BMI category)	Perceived body image (weight)				Test of significance
	Too thin (Underweight) Frequency (%)	Normal (Normal) Frequency (%)	A bit fat (Overweight) Frequency (%)	Too fat (Obese) Frequency (%)	
**Normal** (n = 111)	32 (28.8)	79 (71.2)	0 (0)	0 (0)	c^2^ = 45.03, p< 0.001
**Overweight** (n = 105)	0 (0)	53 (50.5)	37 (35.2)	15 (14.3)	
**Obese** (n = 69)	0 (0)	8 (11.6)	21 (30.4)	40 (58.0)	

Measure of agreement (Kappa) = 0.341, p < 0.001

### Participants’ weight control practices

**Figure 2:**
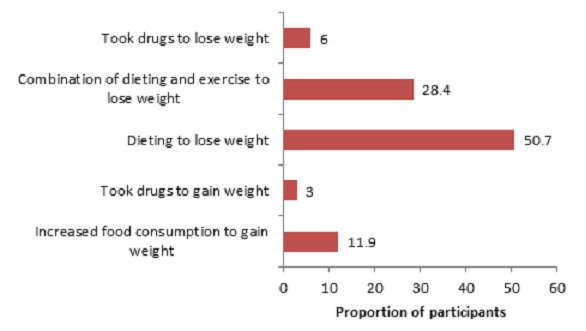
Participants’ weight control practices

Only 67 (23.5%) of the 285 participants were engaged in weight control practices. Of these, 8 (11.9%) and 2 (3.0%) eat more and used drugs respectively to gain weight; while 34 (50.7%), 19 (28.4%) and 4 (6.0%) eat less, employed a combination of dieting and exercise, and used drugs respectively to lose weight (Figure 2).

## Discussion

The preponderance of participants in their third decade of life (30-39 years) in this study could be due to the fact that they are employees of private establishments that are more likely to employ young people (to maximize productivity). Similarly, the preponderance of males (particularly those with tertiary education) could be due to the low female enrolment into basic formal education in Sokoto (with a male to female ratio of 2:1), thus making males for eligible for employment compared to females [37]. The high prevalence of overweight (36.8%) and obesity (24.2%) among the participants in this study compares well with the 37.8% and 17.8% prevalence of overweight and obesity respectively in a study among financial institution workers in Accra, Ghana [38]. Noticeably, the prevalence of obesity among the participants in this study is almost at par with that of workers in the United States (27.7%) [39]. It is therefore evident that obesity has assumed epidemic proportion globally, and not just a problem of developed countries. The significantly higher prevalence of overweight among males (males 43.5%, females 28.2%) and obesity among females (males 19.3%, females 29.0%) in this study corroborates the findings in a study among traders in Ijebu Ode, Nigeria, that reported higher prevalence of overweight in males (males 21.7%, females 20.4%) and obesity in females (males 29.4%, females 33.6%) [40]. These findings suggest the need to pay attention to sex differences in the design of weight control interventions.

Weight misperceptions were prevalent among the participants in this study particularly among those with overweight (64.8%) and obesity (42.0%), and there was poor agreement between actual and perceived weight (k statistics = 0.341, p < 0.001). Similar to the findings in this study, a study by Salem et al, reported 91.0% prevalence of misperception among overweight participants [41]. Another study by Bhanji et al, reported 50.0% and 73.0% prevalence of misperception among overweight and obese participants respectively [20]. In contrast to the findings in this study, a study by Iliyasu et al, reported higher prevalence of misperception among participants in the two extremes of underweight (84.1%) and overweight (84.2%), with 15.5% of participants considering obesity as socially desirable and a sign of good living and affluence [32]. These findings have serious implications in view of the adverse psychosocial outcomes associated with misperception of body weight status [15]. In addition to the documented evidence of the perception of being “too thin” (i.e., underweight) or “too fat” (i.e., obese) being the best predictor of problem behavior [42], it has been found that, regardless of body mass index, extreme perceptions of weight appear to be significant risk factor for suicide behavior [13]. Despite the high prevalence of overweight and obesity (61.0%) among the participants in this study, less than a fifth (23.5%) engaged in weight control practices; this could be related to the high prevalence of misperceptions among them. These findings support the submission of Okon et al, [43] on weight misperception constituting a considerable challenge to obesity control, and they underscore the need for a holistic approach to obesity control interventions that encompasses both body image perception and nutritional assessment.

## Conclusion

This study showed high prevalence of overweight and obesity, weight misperceptions, and poor uptake of weight control practices among office employees in Sokoto, Nigeria. These findings underscore the need for a holistic approach to obesity control interventions that encompasses both body image perception and nutritional assessment.

### What is known about this topic

- The prevalence of overweight and obesity is rising in low- and middle-income countries particularly among groups with largely sedentary occupations;
- The perceived body weight is not always a true representation of the body weight status;
- Accurate perception of body weight status is crucial to uptake of body weight control interventions.

### What this study adds

- The prevalence of obesity is high among office employees in Sokoto, Nigeria, and at par with that of workers in the United States;
- While body weight misperception was very prevalent among the participants, those with normal weight were thrice more likely to perceive their weight accurately compared with those with overweight and obesity;
- This study highlights the low uptake of weight control interventions despite the high prevalence of overweight and obesity among the participants.

## Competing interests

The authors declare no competing interest.
